# Melting Curve Analysis after T Allele Enrichment (MelcaTle) as a Highly Sensitive and Reliable Method for Detecting the *JAK2*V617F Mutation

**DOI:** 10.1371/journal.pone.0122003

**Published:** 2015-03-20

**Authors:** Soji Morishita, Kochi Takahashi, Marito Araki, Yumi Hironaka, Yoshitaka Sunami, Yoko Edahiro, Miyuki Tsutsui, Akimichi Ohsaka, Satoshi Tsuneda, Norio Komatsu

**Affiliations:** 1 Department of Transfusion Medicine and Stem Cell Regulation, Juntendo University Graduate School of Medicine, Tokyo, Japan; 2 Department of Life Science and Medical Bioscience, Waseda University, Tokyo, Japan; 3 Department of Hematology, Juntendo University School of Medicine, Tokyo, Japan; B.C. Cancer Agency, CANADA

## Abstract

Detection of the *JAK2*V617F mutation is essential for diagnosing patients with classical myeloproliferative neoplasms (MPNs). However, detection of the low-frequency *JAK2*V617F mutation is a challenging task due to the necessity of discriminating between true-positive and false-positive results. Here, we have developed a highly sensitive and accurate assay for the detection of *JAK2*V617F and named it melting curve analysis after T allele enrichment (MelcaTle). MelcaTle comprises three steps: 1) two cycles of *JAK2*V617F allele enrichment by PCR amplification followed by *Bsa*XI digestion, 2) selective amplification of the *JAK2*V617F allele in the presence of a bridged nucleic acid (BNA) probe, and 3) a melting curve assay using a BODIPY-FL-labeled oligonucleotide. Using this assay, we successfully detected nearly a single copy of the *JAK2*V617F allele, without false-positive signals, using 10 ng of genomic DNA standard. Furthermore, MelcaTle showed no positive signals in 90 assays screening healthy individuals for *JAK2*V617F. When applying MelcaTle to 27 patients who were initially classified as *JAK2*V617F-positive on the basis of allele-specific PCR analysis and were thus suspected as having MPNs, we found that two of the patients were actually *JAK2*V617F-negative. A more careful clinical data analysis revealed that these two patients had developed transient erythrocytosis of unknown etiology but not polycythemia vera, a subtype of MPNs. These findings indicate that the newly developed MelcaTle assay should markedly improve the diagnosis of *JAK2*V617F-positive MPNs.

## Introduction

In polycythemia vera (PV), essential thrombocythemia (ET), and primary myelofibrosis (PMF), the major subtypes of classical myeloproliferative neoplasms (MPNs), patients possess a common somatic point mutation in *JAK2* that results in the conversion of valine 617 to phenylalanine (V617F) [1–3]. *JAK2*V617F is a gain-of-function mutation that causes the constitutive activation of *JAK2* and plays a causal role in MPNs development *in vivo* [4]. The detection of *JAK2*V617F is one of the major criteria for the diagnosis of MPNs according to the 2008 WHO classification [5]; thus, the precise detection of *JAK2*V617F is important for the accurate diagnosis of MPNs.

To date, various *JAK2* genotyping methods, such as Sanger sequencing, pyrosequencing, DNA melting curve analysis, and allele-specific PCR (AS-PCR), have been developed [6–8]. Additionally, our group recently developed alternately binding probe competitive PCR (ABC-PCR) [9]. These methods have similar sensitivities for detecting a target allele, with frequencies from 0.1% to 10%. More recently, two highly sensitive methods that enable the detection of low-frequency *JAK2*V617F have been developed. One of these methods is the AS-PCR assay with restriction enzyme *Bsa*XI digestion to eliminate the *JAK2* wild-type allele, which achieves a sensitivity of 0.001% [10]. However, due to incomplete digestion of the wild-type allele, false-positive results have been reported [11]. The other method is peptide nucleic acid (PNA) clamping PCR analysis, which detects the target mutation at a frequency as low as 0.05% [12]. Because the PNA probe cannot completely block wild-type allele amplification, this assay yields false-positive results in approximately 10% of *JAK2*V617F-negative specimens [12]. Therefore, to further improve the diagnosis of MPNs, a more reliable *JAK2*V617F detection assay is required.

Here, we have established a highly sensitive and reliable assay, which we named melting curve analysis after T allele enrichment (MelcaTle), to detect the *JAK2*V617F mutation. As it can identify nearly a single copy of the *JAK2*V617F mutation without yielding false-positive results, this assay is expected to be useful as an accurate and precise diagnostic tool for MPNs.

## Materials and Methods

### Oligonucleotides

All sequences and positions of the primers and probes that were used are listed in Table 1. All primers were purchased from Life Technologies Corp. The quenching probe (Q-Probe) labeled at its 3’ end with BODIPY FL, the BNA probe, the PNA probe, and the TaqMan probe were purchased from J-Bio 21 Corp., GeneDesign Inc., Panagene Inc., and Life Technologies Corp., respectively. The specificities of the primers and probes were verified by searching the DNA data bank of Japan using BLAST.

**Table 1:** Sequences of primers and probes used in this study.

Name	Description	Sequence (5′–3′)	Tm (°C) ^†^	Position^‡^	Ref.
Primer					
FO	outer forward primer	CACTTTGATCTCCATATTCCAG	60.1	54772–54793	[9]
RO	outer reverse primer	TGCCATAATCTCTTTTGC	57.2	55364–55347	[9]
FI	inner forward primer	ATCTATAGTCATGCTGAAAGTAGGAGAAAG	65.4	54871–54900	[1]
RI-A	primary inner forward primer	CTGAATAGTCCTACAGTGTTTTCAGTTTCA	66.3	55234–55205	[1]
RI-B	secondary inner reverse primer	CATTAGAAAGCCTGTAGTTTTACTTACTC	63.6	55103–55075	This study
FTQ	forward primer for AS-PCR	TGATGAGCAAGCTTTCTCAC	62.0	55011–55030	[9]
FAS	*JAK2*V617F specific forward primer	AGCATTTGGTTTTAAATTATGGAGTATATT	63.3	55032–55061	[9]
Probe					
Q-probe		TCCACAGAAACATACTCC-(BODIPY-FL)	57.5	55069–55052	This study
BNA probe		**CC**A**C**AG*ACA* **C**A**T**A**CT** *	78.0	55068–55054	This study
PNA probe		CCACAGACACACACT	75.6	55068–55054	This study
ASTQ probe		(FAM)-TGTGGAGACGAGAGTAA-(MGB)	57.9	55064–55080	[13]

* Sequences in italic and bold letters are synthesized with locked nucleic acid (LNA) and BNA, respectively.

^†^ The Tm is estimated on the premise that the oligonucleotides hybridize to the complementary sequences.

^‡^ The position is based on the sequence obtained from GenBank Accession No. AL161450.

### Standard DNA templates

Standard DNA templates were generated from either PCR products or genomic DNA. To construct an amplicon-based DNA standard, DNA fragments (594 bp at the locus from 54772 to 55365 in Acc. No. AL161450) containing the wild-type *JAK2* or V617F mutant were first PCR-amplified from genomic DNA using the FO and RO primers. The concentrations of the purified PCR products were measured using a NanoDrop 1000 spectrophotometer (Thermo Scientific). The amplicon-based DNA standard was then prepared by mixing each amplicon to obtain *JAK2*V617F allele frequencies of 0.001%, 0.005%, 0.01%, 0.05%, 0.1%, 0.5%, 1%, and 10%. To construct the genomic DNA standard, genomic DNA was extracted from *JAK2* wild-type UT-7/EPO and *JAK2*V617F homozygous HEL cells [14, 15] using a QIAamp DNA Mini kit (Qiagen) according to the manufacturer’s instructions. The concentration and *JAK2* copy number of each extracted DNA sample were measured using a Quantus Fluorometer (Promega) and the QuantStudio 3D digital PCR system (Life Technologies), respectively. Genomic DNA standards with *JAK2*V617F allele frequencies of 0%, 0.01%, 0.05%, 0.5%, and 1% were prepared by mixing the proper amounts of each type of genomic DNA. All of the genomic DNA standards were prepared at the following concentrations: 10, 50, and 100 ng/μL.

### Human specimens

Genomic DNA from 38 MPNs patients visiting Juntendo University Hospital (Hongo, Tokyo, Japan) and 30 healthy volunteers was used. The study was conducted in accordance with the Declaration of Helsinki and was approved by the ethics committee of the Juntendo University School of Medicine (IRB#2012135), and written informed consent was obtained from all of the participants prior to sample collection. Genomic DNA was extracted from the peripheral blood of each patient or healthy volunteer using a QIAamp DNA Mini kit (Qiagen) according to the manufacturer’s instructions. The DNA concentrations were measured using a NanoDrop 1000 spectrophotometer (Thermo Scientific), and the DNA was stored at −80°C until use.

### Melting curve analysis after T allele enrichment (MelcaTle)

**Fig 1:**
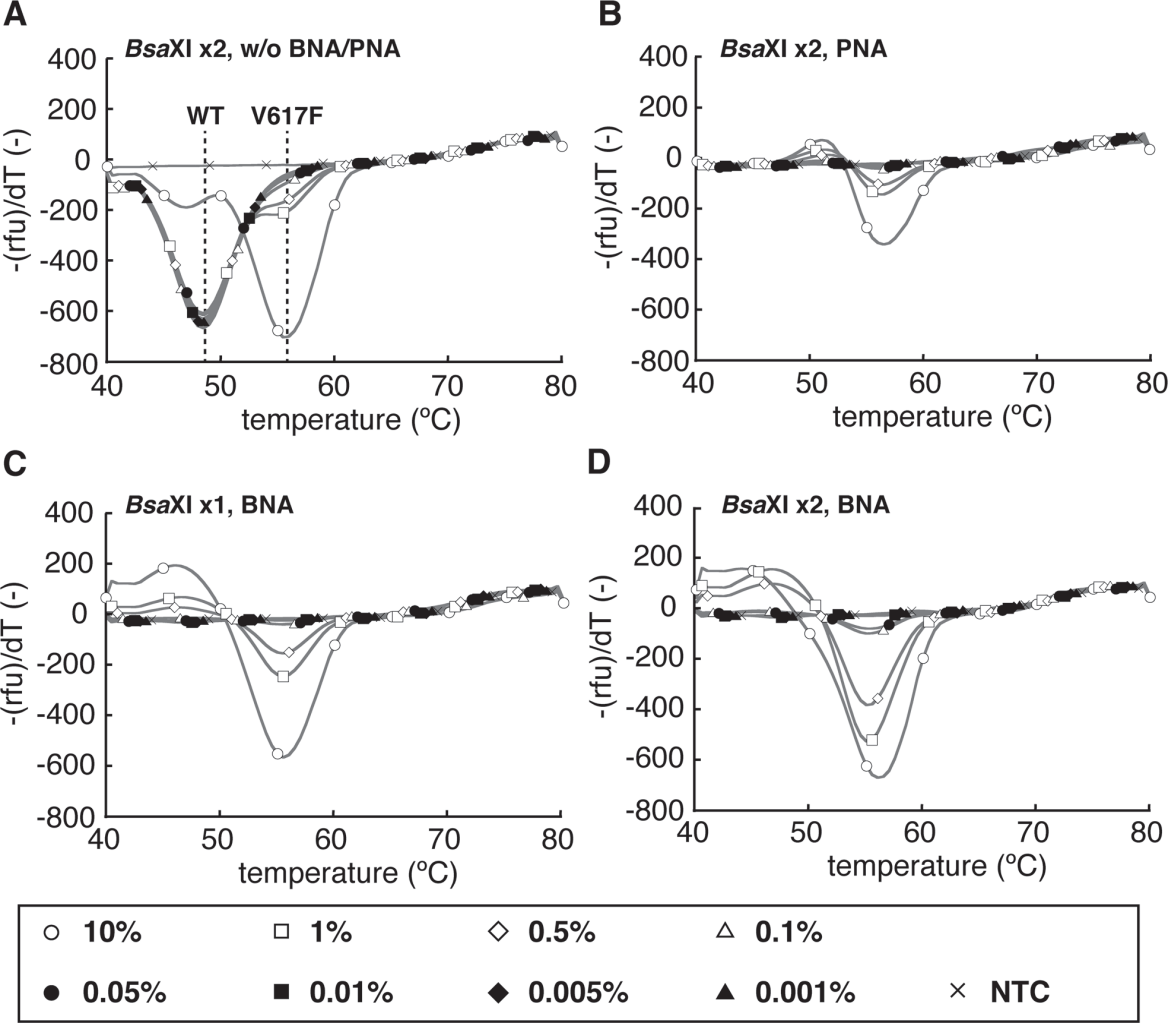
Melting curve profiles obtained by MelcaTle. Samples with known *JAK2*V617F allele frequencies (open circles, 10%; open squares, 1%; open diamonds, 0.5%; open triangles, 0.1%; solid circles, 0.05%; solid squares, 0.01%; solid diamonds, 0.005%; solid triangles, 0.001%) were used as standards. Cross symbols represent no template controls (NTC). A: Melting curve profile obtained without using any clamping probes. B: Melting curve profile obtained using the PNA probe. C and D: Peak profiles after enrichment of the *JAK2*V617F allele by *Bsa*XI digestion once (C) and twice (D). All of the experiments were repeated three times, and the standard deviations of the batches were, at most, 23.2% of the mean of the corresponding peak height. The *P* values of the peak heights against the baseline were < 0.0001.

The initial PCR mixture (20 μL) contained 0.5× Titanium Taq DNA polymerase (TaKaRa Bio), 1× Titanium buffer, 0.2 mM of each dNTP, 0.5 μM each of FO and RO, and 1 μL of template DNA. The PCR conditions were as follows: an initial denaturation step at 94°C for 3 min; 25 cycles of denaturation at 94°C for 30 s, annealing at 58°C for 30 s, and extension at 72°C for 40 s; and a final extension step at 72°C for 2 min. The PCR products (1 μL) were then digested in a reaction mixture (10 μL) with 0.4 units of *Bsa*XI (New England BioLabs) at 37°C for 2 h. The digested PCR products were subjected to another round of PCR using the FI and RI-A primers instead of the FO and RO primers. The second round of PCR products was digested with *Bsa*XI, and then 1 μL of the second-digestion samples were subjected to a final PCR to selectively amplify the *JAK2*V617F allele in a reaction mixture (15 μL) containing 0.5× Titanium Taq DNA polymerase (TaKaRa Bio), 1× Titanium buffer, 0.2 mM of each dNTP, 1.0 μM FI, 0.1 μM RI-B, 0.5 μM BNA probe, and 0.05 μM Q-Probe. The final PCR conditions were as follows: an initial denaturation step at 95°C for 5 min, and 40 cycles of denaturation at 94°C for 30 s, annealing at 60°C for 30 s, and extension at 68°C for 30 s. Finally, the PCR products were subjected to melting curve analysis. The reaction mixtures were incubated at 95°C for 2 min, 65°C for 2 min, and 40°C for 2 min and were then gradually warmed to 80°C at a ramp rate of 0.5°C/s with continuous fluorescence acquisition. The melting curves were created by plotting the differential fluorescence intensity vs the temperature. All of the reactions were performed in triplicate, and the no-template control (NTC) contained sterilized water instead of the template DNA. The first and second rounds of PCR and the *Bsa*XI digestion were performed using a LifePro thermal cycler (Bioer Technology), and the melting assay, including the final PCR, was performed using a CFX96™ real-time system (BioRad). Prior to establishing the MelcaTle protocol, the final PCR and subsequent melting curve analysis was performed in the absence of the clamping probe (Fig. 1A) or in the presence of the PNA probe instead of the BNA probe (Fig. 1B). When using MelcaTle, *JAK2*V617F positivity was defined as the mean peak value + 3σ of the signals obtained from any samples that were lower than the mean value—3σ of the signals from the negative control (100% wild-type *JAK2* allele).

### Allele-specific PCR (AS-PCR)

AS-PCR was performed as previously described [9]. Briefly, quantitative PCR targeting all of the *JAK2* alleles or the *JAK2*V617F allele was performed twice using the FTQ and RI-A or the FAS and RI-A primers. The thermal cycling profile was as follows: 50°C for 2 min, an initial denaturation at 95°C for 10 min, and 50 cycles of denaturation at 95°C for 40 s, annealing at 58°C for 40 s, and extension at 72°C for 40 s. AS-PCR was performed in triplicate for each template DNA. The results were analyzed using CFX Manager software (BioRad).

## Results

### Establishment of MelcaTle

To establish a highly sensitive and reliable method to detect the *JAK2*V617F mutation, we first employed the *Bsa*XI restriction enzyme to eliminate the *JAK2* wild-type allele. This enzyme recognizes GGAGtatgtGT (capital letters: *Bsa*XI recognition sequence), which exists in the coding sequence of wild-type *JAK2* but not of the *JAK2*V617F mutant. First, we amplified DNA harboring the *JAK2*V617F mutant allele at various frequencies from amplicon-based standard DNA using PCR, and then the PCR products were digested with *Bsa*XI (see Materials and Methods). After another round of PCR amplification using a new set of nested PCR primers (Table 1) and subsequent *Bsa*XI digestion, the digested DNA was subjected to a final PCR. Then, the PCR products were subsequently analyzed by melting curve analysis using an oligonucleotide singly labeled with BODIPY-FL (see Materials and Methods). Although we detected a peak representing the *JAK2*V617F mutant allele, a larger peak corresponding to the wild-type allele was also found (Fig. 1A). In the case of samples in which the mutant allele frequencies were lower than 0.1%, the peak corresponding to the wild-type allele overwhelmed that corresponding to the mutant allele, thus decreasing the detection specificity and sensitivity.

**Fig 2:**
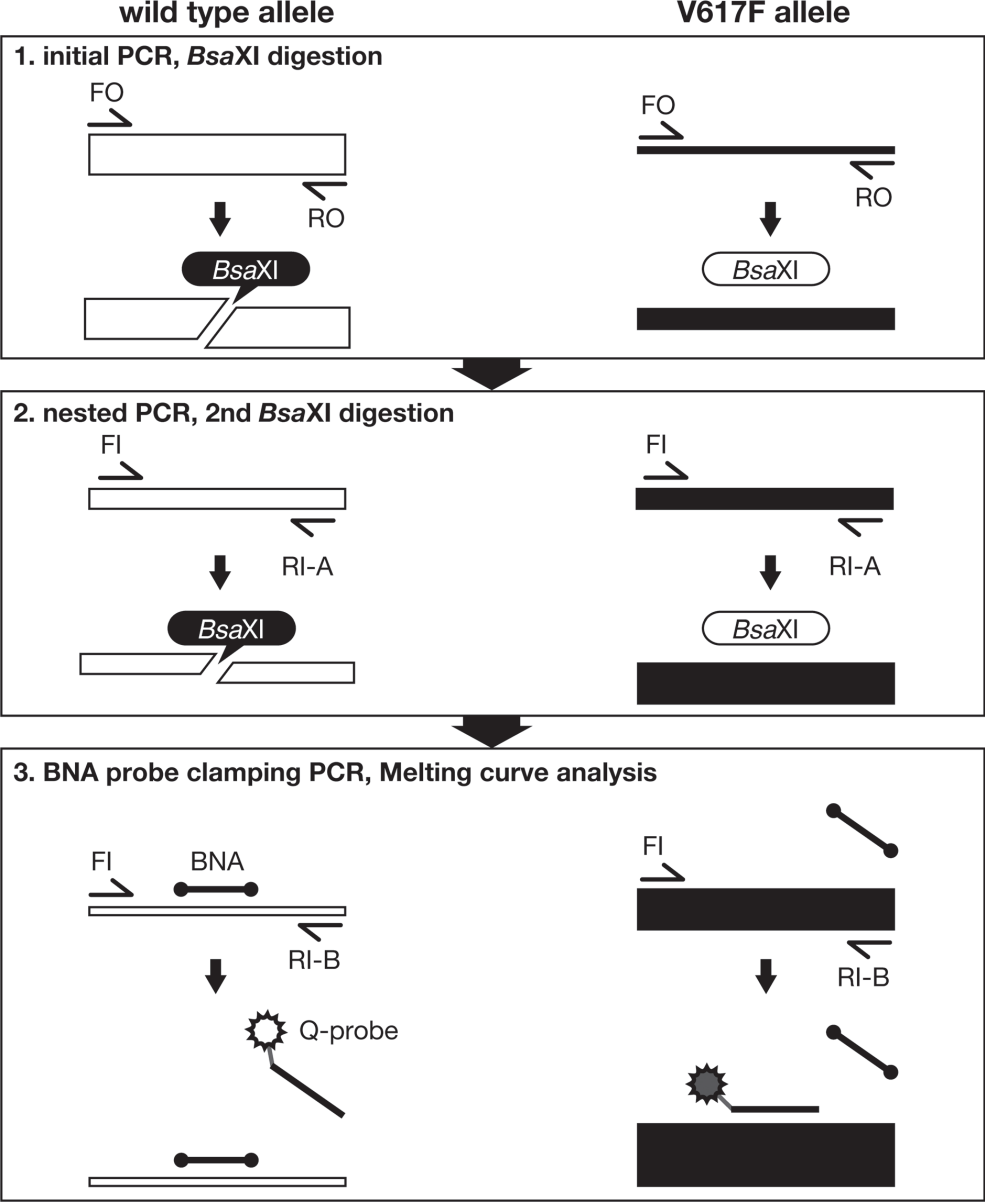
Schematic illustration of MelcaTle. MelcaTle comprises the following steps: 1) PCR amplification of genomic DNA that includes the G1849 region, followed by *Bsa*XI treatment to digest the *JAK2* wild-type allele (G allele), 2) a second nested-PCR amplification and *Bsa*XI digestion to decrease the wild-type allele concentration, and 3) a third PCR using a blocking probe (BNA probe) and subsequent melting curve analysis with a mutant detection probe (Q-probe). The BNA probe inhibits the amplification of the residual G allele that is not digested by *Bsa*XI. The BNA clamping probe also blocks Q-probe annealing to the wild-type (G) allele. The thickness of the bar represents the extent of T allele enrichment during the process.

To overcome this difficulty, we first used a clamping probe prepared from PNA [12, 16, 17] to selectively block undigested wild-type allele DNA during the final PCR and melting curve analysis. The PNA clamping markedly improved the detection of the *JAK2* mutant allele DNA in the melting analysis by eliminating the wild-type peak (Fig. 1B); however, the mutant allele detection sensitivity did not increase with the use of the PNA probe. To screen artificial nucleotides for better clamping efficiency, we tried using BNA. As shown in Fig. 1C, BNA probes could more sensitively detect the *JAK2*V617F mutant allele in the melting curve analysis. Repeated *Bsa*XI digestion was required to detect the mutant allele at the lowest frequency of 0.05% (Fig. 1D), but any further digestion did not improve the lower detection limit (data not shown). Finally, we established a highly sensitive *JAK2*V617F allele detection system (MelcaTle) with virtually no false-positive results (see next section), the schematic presentation of which is shown in Fig. 2.

To further examine the sensitivity of MelcaTle in *JAK2*V617F detection, we applied this assay to genomic DNA standards (see Materials and Methods). MelcaTle successfully detected the *JAK2*V617F mutation at a frequency as low as 0.01% when using 10 ng of the genomic DNA standard, in which only a single copy of the *JAK2*V617F allele was estimated to exist (S1 Fig.). To demonstrate that MelcaTle is able to detect a single copy of the target, we applied 5 ng of genomic DNA standard, which has a *JAK2*V617F allele frequency of 0.01% and in which 1/2 of a copy of the *JAK2*V617F mutant allele is thought to theoretically exist, to the assay. MelcaTle was able to detect *JAK2*V617F positivity in 4 of 10 replicates (S2 Fig.). Thus, MelcaTle has the potential to detect a single copy of *JAK2*V617F from human genomic DNA specimens.

### No false positivity in healthy controls detected by MelcaTle

To validate the diagnostic accuracy of MelcaTle in the detection of *JAK2*V617F, genomic DNA samples from 30 healthy donors (DNA concentrations between 26.1 and 223.5 ng, *abs*260/*abs*280 ≥1.6) were examined. After performing MelcaTle three times for each sample (a total of 90 reactions), we did not detect any peak corresponding to the *JAK2*V617F mutant allele (S3 Fig.), implying that MelcaTle did not give rise to any false-positive results. According to a previous study, conventional AS-PCR can generate false-positive results when screening healthy individuals [8]. Thus, to the best of our knowledge, MelcaTle is an excellent assay for detecting nearly a single copy of the *JAK2*V617F mutation from a minimal amount (10 ng) of genomic DNA without yielding any false positivity.

### MelcaTle is a more reliable diagnostic tool

**Fig 3:**
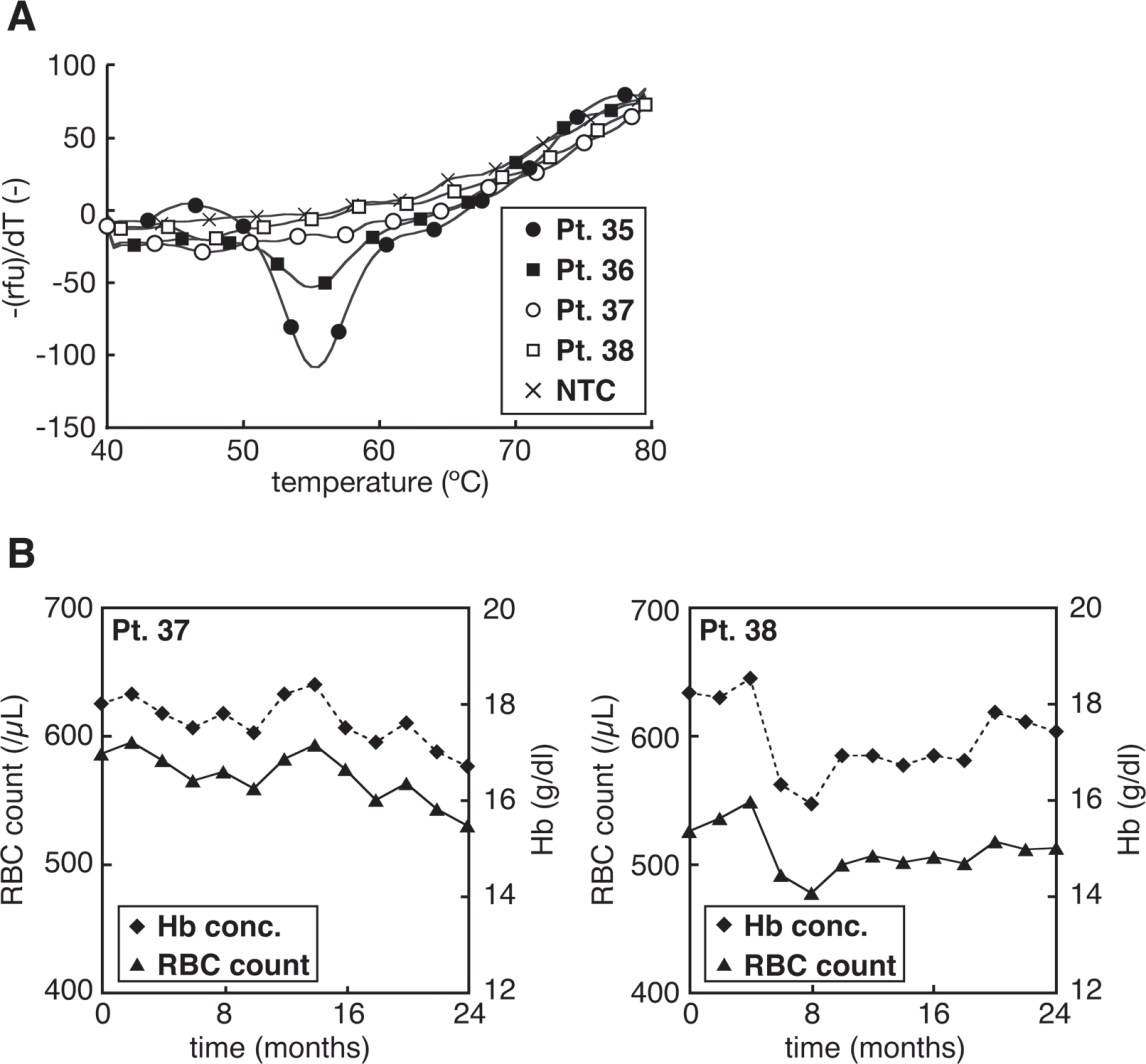
The application of MelcaTle to human specimens. A: Melting curve profiles of MPNs-suspected patient specimens exhibiting discrepant results between MelcaTle and AS-PCR. MelcaTle and other clinical data defining ET (solid symbols) or secondary erythrocytosis (open symbols). B: Transition of the RBC count and hemoglobin concentration in patients with secondary erythrocytosis. The symbols represent the hemoglobin concentration (diamonds) and RBC count (triangles).

Upon establishing MelcaTle for the more sensitive detection of the *JAK2*V617F mutant allele, we screened the genomic DNA of 38 MPNs-defined or MPNs-suspected patients (DNA concentrations between 17.0 and 168.8 ng, *abs*260/*abs*280 ≥1.6) whose *JAK2*V617F mutation was previously identified by AS-PCR (Table 2). In a previous study [18], the positivity threshold for *JAK2*V617F in AS-PCR was set at 1.0%. In two patients in the present study (patients #35 and 36), MelcaTle but not AS-PCR detected the *JAK2*V617F mutation (Table 2, Fig. 3A solid symbols), indicating that these patients were strongly suspected to have MPNs. In agreement with this finding, the bone marrow of these patients showed large and mature megakaryocyte proliferation with persistent thrombocytosis. Therefore, these patients were diagnosed as having ET according to WHO2008 criteria [5], demonstrating the higher diagnostic sensitivity of MelcaTle.

**Table 2:** The presence of *JAK2*V617F mutation in the patients with MPNs.

Pts. No.	Sex	MelcaTle	AS-qPCR (%)*	Pts. No.	Sex	MelcaTle	AS-qPCR (%)*
**1**	M	+	100	**20**	M	+	2.5
**2**	F	+	100	**21**	M	+	1.8
**3**	M	+	80	**22**	M	+	1.7
**4**	M	+	79.9	**23**	M	+	1.5
**5**	M	+	60.4	**24**	M	+	1.5
**6**	M	+	59.2	**25**	M	+	1
**7**	F	+	42.5	**26**	M	−	0.9
**8**	F	+	42	**27**	M	−	0.8
**9**	F	+	23.4	**28**	F	−	0.8
**10**	F	+	22.1	**29**	M	−	0.7
**11**	M	+	10.6	**30**	F	−	0.7
**12**	M	+	10	**31**	M	−	0.6
**13**	M	+	9.5	**32**	M	−	0.6
**14**	M	+	9.3	**33**	M	−	0.5
**15**	F	+	9.1	**34**	M	−	0.5
**16**	F	+	8.1	**35**	M	+	0.8
**17**	F	+	7.1	**36**	M	+	0.7
**18**	M	+	6	**37**	M	−	4.7
**19**	M	+	5.2	**38**	M	−	1.4

* *JAK2*V617F mutation is presented as the percentage of *JAK2*V617F mutation with respect to the entire *JAK2* genome. The threshold of *JAK2*V617F positivity is defined as 1% in AS-PCR.

Conversely, MelcaTle identified patients #37 and 38 as negative for the *JAK2*V617F mutation, whereas AS-PCR identified them as positive (Fig. 3A open symbols and Table 2). Because these two male patients presented relatively high hemoglobin concentrations (18.0 and 18.2 g/dl, respectively) at the time of genomic DNA isolation and were identified as *JAK2*V617F positive by AS-PCR, these patients were suspected as having PV. However, during the follow-up, the hemoglobin concentrations and red blood cell counts of the patients gradually decreased (Fig. 3B), and based on clinical observations, the patients likely had secondary erythrocytosis. These observations indicated that MelcaTle is a more accurate method than AS-PCR for diagnosing the *JAK2*V617F mutation.

## Discussion

In this study, we have established MelcaTle as a sensitive and reliable *JAK2*V617F detection method comprising three elements: 1) reduction of the wild-type allele by *Bsa*XI digestion, 2) blockade of PCR amplification from the residual wild-type alleles by clamping with a BNA probe, and 3) detection of the *JAK2*V617F-specific peak in the melting assay using a Q-Probe [19, 20]. MelcaTle detects the *JAK2*V617F mutation in the target regardless of *JAK2* wild-type allele amplification because peaks corresponding to the *JAK2*V617F are easily distinguished from those corresponding to the wild-type *JAK2*. Furthermore, MelcaTle detects nearly a single copy of the *JAK2*V617F mutation without false-positive results, even with the use of a minimal amount of genomic DNA (10 ng) as a template (S1 and S2 Figs.).

The detection sensitivity of melting curve-based assays, including MelcaTle, may decrease to some extent in cases for which the amplification of the wild-type allele occurs; this is because the peak corresponding to the *JAK2*V617F mutation partially overlaps with that corresponding to the wild-type *JAK2* allele (Fig. 1A). Therefore, the absolute blocking of the amplification of the wild-type allele by clamping is one of the key elements in MelcaTle. Although PNA probes were successfully used as clamping probes in previous assay systems [12, 17], this was not the case in MelcaTle (Fig. 1B). This is presumably due to the formation of various Hoogsteen base-pairing “invasive” triplexes or quadruplexes between PNA and DNA [21] and to the subsequent decrease in the quenching efficiency of BODIPY-FL on the Q-Probe, resulting in low detection sensitivity. In contrast, the BNA probe binds to only the major groove of double-stranded DNA and does not affect the distance between the DNA and the Q-Probe [22]. This explains why the BNA probe more sensitively detected the *JAK2*V617F allele in MelcaTle (Fig. 1D). Although we successfully blocked wild-type allele amplification using the BNA probe, we still need to consider the appearance of some unexpected mutations due to PCR errors or single-nucleotide polymorphisms that alter the accessibility of the BNA probe to wild-type *JAK2* and subsequently allow for the amplification of detectable levels of wild-type *JAK2*. Nevertheless, MelcaTle yielded no positivity in the tests of healthy controls (total 90 tests) (S3 Fig.), demonstrating the diagnostic accuracy of *JAK2*V617F detection.

Although no errors in the experiments conducted in this study were identified, multiple manipulations in MelcaTle would increase the risks of PCR errors and cross contamination compared with simpler and highly sensitive methods such as the MutaQuant assay (Qiagen), which detects the *JAK2*V617F allele at a frequency of 0.01% [23]. To minimize these risks, further improvements of MelcaTle, for example, by using a high-fidelity DNA polymerase instead of Titanium Taq DNA polymerase and by replacing *Bsa*XI, which exhibited inefficient digestion, with genome-editing technologies such as zinc-finger nucleases [24], would be required. These improvements would lead to the establishment of a more practical and powerful assay system for the detection not only of *JAK2*V617F but also of other mutations. To the best of our knowledge, MelcaTle is the most sensitive assay for detecting the *JAK2*V617F mutation at the single-copy level (S2 Fig.), and this method is semi-quantitative when analyzing the signal height in the melting curve analysis (Figs. 1 and S1).

The minimal frequency of the *JAK2*V617F allele that is needed for diagnosing MPNs remains controversial [25]; this was studied using AS-PCR [26, 27]. Applying such an analytically sensitive and diagnostically accurate assay to patients exhibiting low *JAK2*V617F allele frequencies may improve our understanding of the pathogenesis of MPNs. Indeed, we have demonstrated the clinical value of MelcaTle by redefining the *JAK2*V617F mutation status in patients who were diagnosed using AS-PCR (Fig. 3). Furthermore, the need for highly sensitive methods for monitoring the minimal level of residual tumor cells after bone marrow transplantation in MPNs patients is highlighted [28]. Although we do not know the extent to which we need to detect the *JAK2*V617F allele in such MPNs cases, MelcaTle or a modified version of MelcaTle would provide an option for diagnosing the existence of the *JAK2*V617F allele with high accuracy and sensitivity in the future.

## Supporting Information

S1 Fig MelcaTle identifies the *JAK2*V617F allele at a frequency of 0.01% from a minimal amount of DNA sample. Genomic DNAs derived from UT-7/EPO (*JAK2* wild-type allele) and HEL (homologous *JAK2*V617F allele) cells were mixed to obtain genomic DNA standards containing *JAK2*V617F mutations with allele frequencies of 1% (open circles), 0.5% (open squares), 0.05% (open diamonds), 0.01% (open triangles), or 0% (solid circles). Ten nanograms (panel A), 50 ng (panel B), and 100 ng (panel C) of each of the genomic DNA standards were prepared at these concentrations and then applied to MelcaTle. Cross symbols indicate no template controls (NTC).
(PDF)

S2 Fig MelcaTle can detect nearly a single copy of the *JAK2*V617F mutant allele. Theoretically, 1/2 a copy of the *JAK2*V617F mutation per aliquot was used as the starting material. The experiment was performed in 10 replicates (R1 to R10). The relationship between the fluorescence intensity (y-axis) and temperature (x-axis) was plotted. Based on the P-values (<0.0001) and the 99.9% confidence interval using the Tukey-Kramer test, 4 (R2, R4, R7, and R8) of the 10 replicates were identified as *JAK2*V617F-positive.
(PDF)

S3 Fig The application of MelcaTle to healthy individuals. No false positivity was identified in three independent assays using samples from 30 healthy volunteers. The relationship between the fluorescence intensity (y-axis) and temperature (x-axis) was plotted. All of the reactions were repeated three times. PC, positive control; NTC, no template control.
(PDF)
