# Supplementary material for: Melting Curve Analysis after T Allele Enrichment (MelcaTle) as a Highly Sensitive and Reliable Method for Detecting the JAK2V617F Mutation
Source: PLoS One. 2015 Mar 20;10(3):e0122003. doi: 10.1371/journal.pone.0122003 (PMC4368779; doi:10.1371/journal.pone.0122003)
Supplement: S1 Fig — Genomic DNAs derived from UT-7/EPO (JAK2 wild-type allele) and HEL (homologous JAK2V617F allele) cells were mixed to obtain genomic DNA standards containing JAK2V617F mutations with allele frequencies of 1% (open circles), 0.5% (open squares), 0.05% (open diamonds), 0.01% (open triangles), or 0% (solid circles). Ten nanograms (panel A), 50 ng (panel B), and 100 ng (panel C) of each of the genomic DNA standards were prepared at these concentrations and then applied to MelcaTle. Cross symbols indicate no template controls (NTC). (PDF) [file pone.0122003.s001.pdf]

S1 Fig.

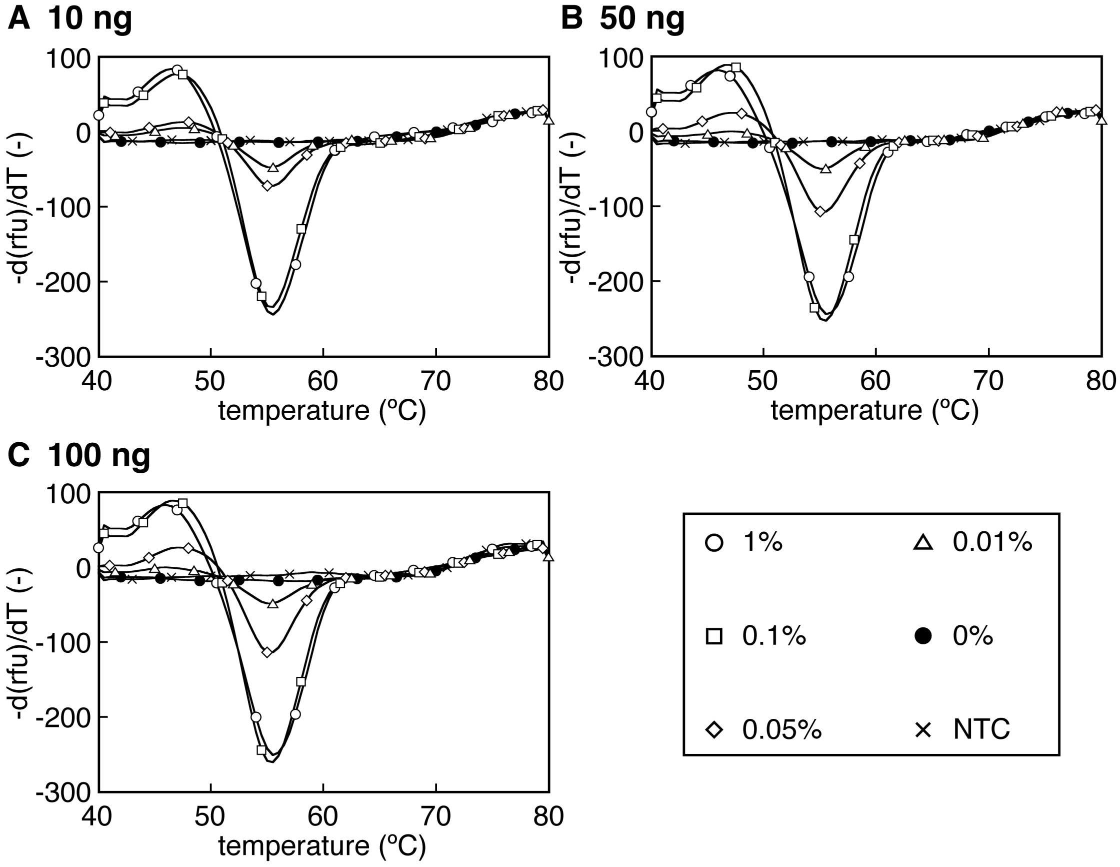

S1 Fig. MelcaTle identifies the *JAK2*V617F allele at a frequency of 0.01% from a minimal amount of DNA sample. Genomic DNAs derived from UT-7/EPO (*JAK2* wild-type allele) and HEL (homologous *JAK2*V617F allele) cells were mixed to obtain genomic DNA standards containing *JAK2*V617F mutations with allele frequencies of 1% (open circles), 0.5% (open squares), 0.05% (open diamonds), 0.01% (open triangles), or 0% (solid circles). Ten nanograms (panel A), 50 ng (panel B), and 100 ng (panel C) of each of the genomic DNA standards were prepared at these concentrations and then applied to MelcaTle. Cross symbols indicate no template controls (NTC).
