# Supplementary material for: Melting Curve Analysis after T Allele Enrichment (MelcaTle) as a Highly Sensitive and Reliable Method for Detecting the JAK2V617F Mutation
Source: PLoS One. 2015 Mar 20;10(3):e0122003. doi: 10.1371/journal.pone.0122003 (PMC4368779; doi:10.1371/journal.pone.0122003)
Supplement: S2 Fig — Theoretically, 1/2 a copy of the JAK2V617F mutation per aliquot was used as the starting material. The experiment was performed in 10 replicates (R1 to R10). The relationship between the fluorescence intensity (y-axis) and temperature (x-axis) was plotted. Based on the P-values (<0.0001) and the 99.9% confidence interval using the Tukey-Kramer test, 4 (R2, R4, R7, and R8) of the 10 replicates were identified as JAK2V617F-positive. (PDF) [file pone.0122003.s002.pdf]

**S2 Fig.**

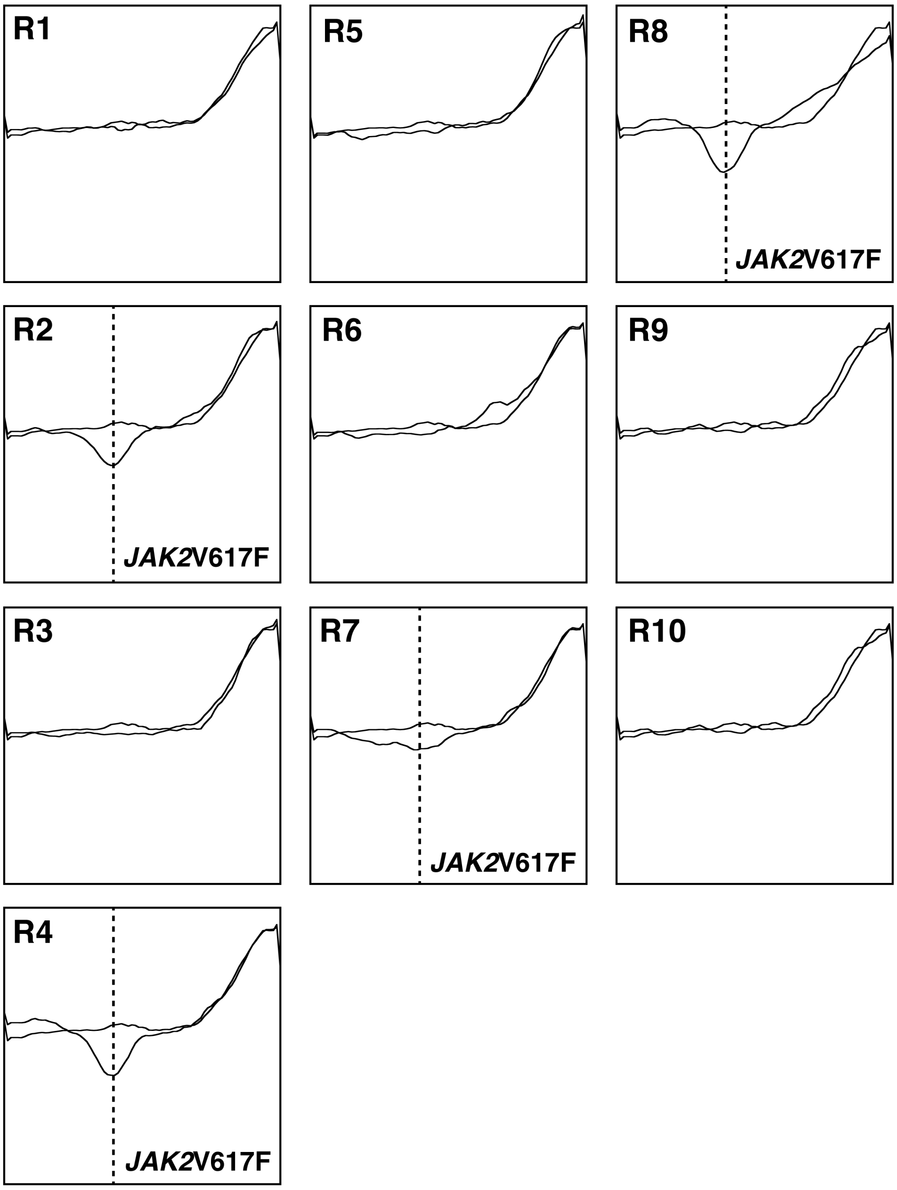


**S2 Fig. MelcaTle can detect nearly a single copy of the *JAK2*V617F mutant allele.** Theoretically, 1/2 a copy of the *JAK2*V617F mutation per aliquot was used as the starting material. The experiment was performed in 10 replicates (R1 to R10). The relationship between the fluorescence intensity (y-axis) and temperature (x-axis) was plotted. Based on the P-values (<0.0001) and the 99.9% confidence interval using the Tukey-Kramer test, 4 (R2, R4, R7, and R8) of the 10 replicates were identified as *JAK2*V617F-positive.
