# Supplementary material for: Melting Curve Analysis after T Allele Enrichment (MelcaTle) as a Highly Sensitive and Reliable Method for Detecting the JAK2V617F Mutation
Source: PLoS One. 2015 Mar 20;10(3):e0122003. doi: 10.1371/journal.pone.0122003 (PMC4368779; doi:10.1371/journal.pone.0122003)
Supplement: S3 Fig — No false positivity was identified in three independent assays using samples from 30 healthy volunteers. The relationship between the fluorescence intensity (y-axis) and temperature (x-axis) was plotted. All of the reactions were repeated three times. PC, positive control; NTC, no template control. (PDF) [file pone.0122003.s003.pdf]

S3 Fig.

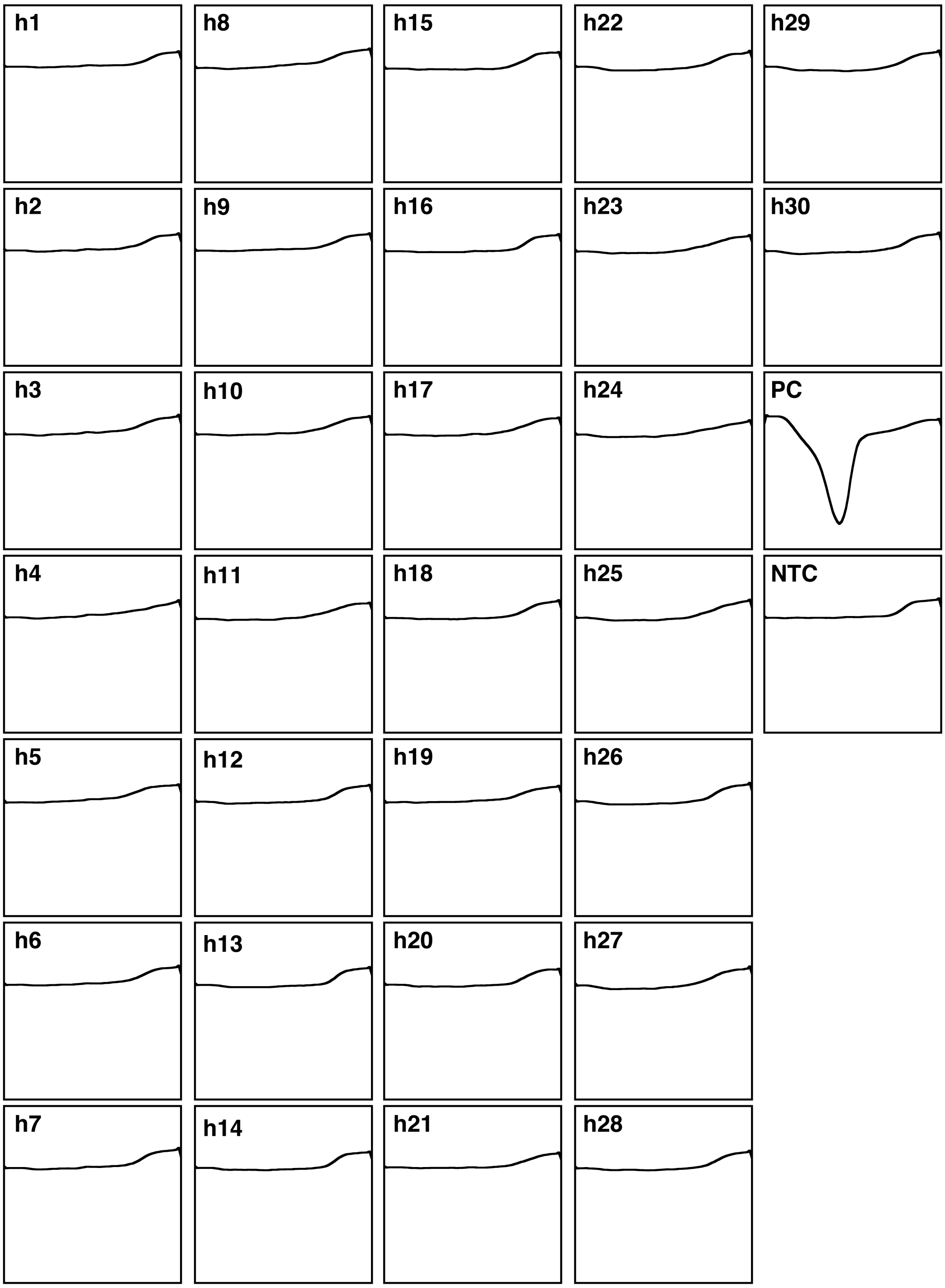

S3 Fig. The application of MelcaTle to healthy individuals. No false positivity was identified in three independent assays using samples from 30 healthy volunteers. The relationship between the fluorescence intensity (y-axis) and temperature (x-axis) was plotted. All of the reactions were repeated three times. PC, positive control; NTC, no template control.
